# Seed Priming with Brassinosteroids Alleviates Chromium Stress in Rice Cultivars via Improving ROS Metabolism and Antioxidant Defense Response at Biochemical and Molecular Levels

**DOI:** 10.3390/antiox10071089

**Published:** 2021-07-07

**Authors:** Farwa Basit, Min Chen, Temoor Ahmed, Muhammad Shahid, Muhammad Noman, Jiaxin Liu, Jianyu An, Abeer Hashem, Al-Bandari Fahad Al-Arjani, Abdulaziz A. Alqarawi, Mashail Fahad S. Alsayed, Elsayed Fathi Abd_Allah, Jin Hu, Yajing Guan

**Affiliations:** 1 Institute of Crop Sciences, College of Agriculture and Biotechnology, Zhejiang University, Hangzhou 310058, China; 11916138@zju.edu.cn (F.B.); 11616036@zju.edu.cn (M.C.); liujiaxin@zju.edu.cn (J.L.); anjianyu@live.cn (J.A.); jhu@zju.edu.cn (J.H.); 2 State Key Laboratory of Rice Biology, Institute of Biotechnology, Zhejiang University, Hangzhou 310058, China; temoorahmed@zju.edu.cn (T.A.); nomansiddique834@gmail.com (M.N.); 3 Department of Bioinformatics and Biotechnology, Government College University, Faisalabad 38000, Pakistan; 4 Botany and Microbiology Department, College of Science, King Saud University, P.O. Box 2460, Riyadh 11451, Saudi Arabia; habeer@ksu.edu.sa (A.H.); aarjani@ksu.edu.sa (A.-B.F.A.-A.); malsayed@ksu.edu.sa (M.F.S.A.); 5 Plant Production Department, College of Food and Agricultural Sciences, King Saud University, P.O. Box 2460, Riyadh 11451, Saudi Arabia; alqarawi@ksu.edu.sa (A.A.A.); eabdallah@ksu.edu.sa (E.F.A.)

**Keywords:** antioxidants, brassinosteroids, chromium, heavy metals, rice

## Abstract

This research was performed to explore the vital role of seed priming with a 0.01 µM concentration of brassinosteroids (EBL) to alleviate the adverse effects of Cr (100 µM) in two different rice cultivars. Seed priming with EBL significantly enhanced the germination attributes (germination percentage, germination energy, germination index, and vigor index, etc.), photosynthetic rate as well as plant growth (shoot and root length including the fresh and dry weight) under Cr toxicity as compared to the plants primed with water. Cr toxicity induced antioxidant enzyme activities (SOD, POD, CAT, and APX) and ROS level (MDA and H_2_O_2_ contents) in both rice cultivars; however, a larger increment was observed in YLY-689 (tolerant) than CY-927 (sensitive) cultivar. EBL application stimulatingly increased antioxidant enzyme activities to scavenge ROS production under Cr stress. The gene expression of SOD and POD in EBL-primed rice plants followed a similar increasing trend as observed in the case of enzymatic activities of SOD and POD compared to water-primed rice plants. Simultaneously, Cr uptake was observed to be significantly higher in the water-primed control compared to plants primed with EBL. Moreover, Cr uptake was significant in YLY-689 compared to CY-927. In ultra-structure studies, it was observed that EBL priming relieved the rice plants from sub-cellular damage. Conclusively, our research indicated that seed priming with EBL could be adopted as a promising strategy to enhance rice growth by copping the venomous effect of Cr.

## 1. Introduction

Soil contamination is turning into an alarming situation because of its negative influences on crop productivity. Abiotic stresses can cause >50% reduction in crop yield worldwide [1]. Besides, heavy metal toxicity is more dangerous towards crop production in this era. Chromium (Cr) is the 7th utmost copious heavy metal in the Earth’s crust and it spreads in soil by different industries such as paints, leather, and fertilizer [2]. According to the US Environmental Protection Agency, Cr contamination is a major cause of human carcinoma [2]. Heavy metals, including Cr, become part of the soil in various ways, cause hazardous effects on plant growth and development, and become a major source of human health complications via entrance into the food chain [3]. Moreover, Cr pollution in the soil is extending day-by-day in numerous parts of the world. Almost 30, 896, and 142 metric tons of Cr are released into the air, water, and soil every year, respectively. Cr has various valance states, i.e., 0 to 6, but trivalent chromite (Cr^+3^) and hexavalent chromate (Cr^+6^) are prevalent in the environment. The Cr^+6^ is considered more unstable, extremely peripatetic, and hazardous, especially with a higher pH than Cr^+3^. Latently, the issue of Cr contamination in agriculture is increasing day by day [4]. 

Rice is a vital food source worldwide due to a huge global population’s dependence to fulfill their nutrition requirements with this crop. China is the leading rice producer, consumer, and importer country. Hence, Cr contamination is a more concerning topic in rice-growing areas of China. It is approximated that almost 10% of soil is contaminated with heavy metals in China [5]. Apart from rice, some other plants such as *Hibiscus esculentus* L. [6], *Pisum sativum* L. [7], *Triticum aestivum* L. [8], *Glycine max* L. [9], *Zea mays* L. [10], and *Lycopersicon esculentum* [11] are also being contaminated with Cr. Once, Cr is taken up by plants, nutrient transportation as well as metabolic activities are disturbed, and consequently, crop yield is compromised [5]. For example, uptake of Cr^+6^ causes accumulation and disturbs the uptake of micronutrients such as Mn, Zn, Cu, Fe, etc. 

Moreover, Cr toxicity also induces cellular oxidative stress by triggering the accumulation of reactive oxygen species (ROS), which leads to the necrosis of plants. Several studies have reported that the accumulation of Cr in plants causes severe damage to crop production by increasing toxicity and inhibiting plant growth [12,13,14]. This growth retardation could be linked to the huge disturbance in cellular homeostasis and sub-cellular organelle damage [15,16]. To reduce the ROS activity, plants initiate an efficient mechanism of detoxification by organizing the autoxidation defense mechanism, which consists of superoxide dismutase (SOD), catalase (CAT), peroxidase (POD), and ascorbate peroxidase (APX) as an enzymatic compound as well as glutathione, ascorbic acid, proline, etc. as a non-enzymatic compound. However, plants can build multiple approaches to cope with heavy metal stresses such as Cr, with an increased antioxidant enzyme activity and decreased cellular ROS concentration [13,17,18].

Brassinosteroids (BRs) are considered as the sixth class of plant steroid hormones, which have pleiotropic properties in plants. They can protect plants under various biotic and abiotic stress conditions. Exogenous solicitation of BRs increases stress tolerance in plants [19]. Brassinolide, 28-homobrassinolide (28-HomoBL), and 24-epibrassinolide (EBL) are the three important BRs investigated in various aspects. 24-Epibrassinolide plays an important role in mitigating various plant stresses, including biotic and abiotic stresses. The 24-epibrassinolide is the most biologically active BR compound involved in developmental processes, cell division, elongation, gene expression, and vascular differentiation in plants [20]. It is known to improve plant growth by enhancing the chlorophyll contents, antioxidant enzymes, and up-regulate stress-response gene expression [21]. 

Latent investigations indicate that EBL plays an essential role in mitigating the Cr^+6^ toxicity by stimulating the antioxidant enzyme activities. The present study was further deepened to understand the Cr^+6^ induced sub-cellular damage in rice plants, followed by relieving this stress damage at the physiological and molecular level by applying EBL in a sensitive and tolerant rice cultivar. To the best of our knowledge, this is the first report investigating the comparative physiological and molecular responses of rice plants to the application of EBL in stress-sensitive and tolerant rice cultivars.

## 2. Materials and Methods

### 2.1. Seed Materials and Brassinosteroids (EBL) Preparation

The two rice cultivars, Chunyou 927 (CY-927, sensitive), Yliangyou 689 (YLY-689 tol-erant), were used in this experiment which provided by the Zhejiang Nongke Seeds CO., LTD. Hangzhou, Zhejiang Province, China. 24-Epibrassinolide (EBL) was obtained from the Shanghai Aladdin Biochemical Technology Co., Ltd., China. The BR liquefication was made in an appropriate amount of ethanol and a standard solution (10^−5^ M) concentration was prepared by adding ddH_2_O and 0.05% Tween-20 as a surfactant.

### 2.2. Seed Priming and Germination Test 

For seed priming, rice seeds were firstly immersed in 5% (*w/v*) sodium hypochlorite (NaOCl) solution for 15 min sterilization and gently rinsed with distilled water to remove residual chloride. The sterilized seeds were then primed with 0.01µM EBL at 30 °C in darkness for 24 h, and were dried at room temperature to the seed original moisture con-tent. The seeds primed with water (H2O) were considered as a control.

The seed germination test was carried out after priming and, for this purpose, 50 seeds per box (12 × 18 cm) were germinated with three replications. The germination experiment was carried out in a growth chamber at 25 °C with an alternation cycle of 8 h illumination and 16 h dark conditions for 14 days [22]. Incubated seeds were exposed to different concentrations (0, 50, 100, 200, 300, and 400 µM) of Cr. The selection of Cr concentration for further experimentation was based on this primary experiment, which concluded that 100 µM Cr caused significant damage to plant growth without killing the plants. 

Total germinated seeds (seed radicle visibly protrudes through the seed coat and the radicle reaches to the length of the whole seed) were counted on the 5th day of germination and deliberated as germination energy (G.E). Moreover, the germination percentage (G.P) was recorded on the 14th day. Germination Index (G.I), Mean Germination Time (MGT), as well as Vigor Index (V.I), was measured by following formulas [22].

G.I = Σ(Gt/Tt)(1)

MGT = Σ(Gt × Tt)/ΣGt(2)

V.I = Germination% × [Shoot length + Root length](3)

Gt is the total calculated germinated seeds on day t, and Tt is the time conforming to Gt in days [22].

### 2.3. Plant Growth Conditions 

The incubated seeds were treated with a 100 µM concentration of K_2_Cr_2_O_7_ with a nutrient media solution. The composition of the nutrient solution was 0.5 µM potassium nitrate (KNO_3_), 0.5 µM calcium nitrate (Ca(NO_3_)_2_), 0.5 µM magnesium sulfate (MgSO_4_), 2.5 µM monopotassium phosphate (KH_2_PO_4_), 2.5 µM ammonium chloride (NH_4_Cl), 100 µM ferric EDTA (Fe–K–EDTA), 30 µM boric acid (H_3_BO_3_), 5 µM manganese monosulfate (MnSO_4_), 1 µM copper sulfate (CuSO_4_), 1 µM zinc sulfate (ZnSO_4_), and 1 µM ammonium heptamolybdate ((NH_4_)6Mo_7_O_24_) per liter. The pH of the nutrient solution was adjusted to 5.0 with HCl and NaOH. The concentration was based on findings from a preliminary experiment with various Cr^6+^ (K_2_Cr_2_O_7_) concentrations solutions (0, 50, 100, 200, 300, and 400 µM). The Cr concentrations 50 µM exhibited slight damage to plant growth. Although, a Cr concentration of 100 µM exhibited substantial damage to plant growth; however, concentrations greater than 100 µM were excessively toxic for the growth and killed the plants.

### 2.4. Experimental Design and Treatment Pattern

The experiment was conducted in hydroponic conditions. Two-week-old seedlings (primed with water and with 0.01 µM EBL) were treated with 100 µM Cr concentration and without Cr treatment considered as control (CK). The experiment was conducted through a completely randomized design (CRD) and the boxes were repositioned every day inside the growth chamber. Sampling was carried out at 21 days to perform numerous observations and measurements. 

### 2.5. Plant Growth Investigation

The plants were harvested and immersed in a bucket containing ddH_2_O to remove the remnants of the disinfectant and inspect the safety of the roots. The plants were uprooted, and the lengths of shoots and roots were calculated, including the measurement of fresh mass. To calculate their dry masses, roots and shoots were dried in an oven at 80 °C for 24 h. 

### 2.6. Measurement of Chlorophyll Pigments 

The photosynthetic pigments such as chlorophyll a, b, and total chlorophyll were determined by following the referenced method [23]. In short, fresh leaves (0.2 g) were standardized inside 3 mL ethanol (95%, *v/v*). The centrifugation of homogenate was done at 5000× *g* for 10 min and, subsequently, the supernatant was obtained. Next, 9 mL ethanol (95%, *v/v*) was supplemented with a 1 mL aliquot of the supernatant. Afterward, the measurements were made at the wavelengths of 665 and 649 nm through using an ultraviolet-visible spectrophotometer (UV-1900, Shimadzu, Japan). The following equations were utilized for calculating the pigment contents.

Chlorophyll a (Chla) = 13.95 _A665_ − 6.88 A_649_ (4)

Chlorophyll b (Chlb) = 24.96 A_649_ − 7.32 _A665_ (5)

Total chlorophyll content = Chla + Chlb(6)

The quantities of pigments were expressed as milligrams per liter of plant material.

### 2.7. Measurement of Cr Contents 

Elemental analysis to deliberate Cr contents was accomplished on dried roots and shoots. Dry plant samples (0.2 g) for each treatment were assimilated through 5 mL concentrated HNO_3_ and HCLO_4_ (5:1, *v/v*) in a furnace at 70 °C for nearly 5 h. The dilution of the processed samples was done with 2% HNO_3_ to make an ultimate quantity of 10 mL for Cr content investigation. The filtrate was used to investigate Cr and microelements such as Mn, Cu, and Zn through the use of an atomic absorption spectrophotometer (iCAT-6000-6300, Thermo-Fisher Scientific, Waltham, MA, USA) [24].

### 2.8. Transmission Electron Microscopy Analysis

Shoot sections deprived of veins (8–10) per treatment after 14 days of treatment were obtained from indiscriminately collected seedlings and put into 2.5% (*v/v*) glutaraldehyde in 0.1 M PBS (sodium phosphate buffer with pH 7.4) as well as washed thrice with the alike PBS. Further, the leaves were postfixed in 1% OsO_4_ (osmium (VIII) oxide) for 1 h. After that, it was washed three times in 0.1 M PBS, with 10 min gaps between each wash. After 15–20 min, the leaves were dehydrated within the classified categorization of ethanol (50%, 60%, 70%, 80%, 90%, 95%, and 100%, respectively) and splashed with absolute acetone 20 min. Then, the leaves were soaked in Spurr’s resin for overnight. Consequently, ultrathin sections (80 nm) of samples were cut and placed in copper nets to observe through a transmission electron microscope (JEOLTEM-1230EX) at a hastening voltage of 60.0 kV.

### 2.9. Investigation of Malondialdehyde (MDA) Contents and H_2_O_2_ Production

The measurement of MDA concentration was carried out with 2-thiobarbituric acid (TBA). Approximately, 1.5 mL of extract was homogenized in 2.5 mL of 5% TBA diluted in 5% trichloroacetic acid (TCA). The homogenate sample was heated at 95 °C for 15 min, quickly chilled in ice, and centrifuged at 5000× *g* for 10 min. The absorbance of the supernatant was determined at two wavelengths, 532 and 600 nm, using a UV–vis spectrophotometer (Hitachi U-2910, Tokyo, Japan) [25]. MDA values were expressed as nmol mg^−1^ protein. After absorbance determination, calculations were made according to the following formula.

MDA (nmol g^−1^ FW) = [(OD532 − OD600) × A × V] ÷ (a × E × W)

A = Total Reaction Solution + Enzyme extract

V = Total volume of buffer used for enzyme extract

a = Volume of the enzyme extract used

W = Fresh weight of the sample

E = Constant for MDA (1.55 × 10^−1^)

The reaction mixture without enzyme extract was used as a control and its measurements were subtracted from treatments for accuracy.

To measure hydrogen peroxide (H_2_O_2_), the plant tissues were homogenized in phosphate buffer followed by centrifugation at 6000× *g*. The supernatant was mixed with 0.1% titanium sulfate containing 20% (*v/v*) H_2_SO_4_, followed by centrifugation. The intensity of yellow color was estimated colorimetrically at 410 nm using the above-mentioned unit of the UV–vis spectrophotometer [26]. H_2_O_2_ concentration was calculated by using a standard curve constructed with the known concentrations of H_2_O_2_. A control reaction mixture without the plant tissues was used and its measurements were subtracted from treatments for accuracy. The H_2_O_2_ concentration was calculated in terms of (μmol g^−1^ FW) at 25 ± 2 °C.

### 2.10. Measurement of Antioxidant Enzyme Activity

Vigorous samples (0.5 g) of both shoots and roots were standardized inside 8 mL of 50 mM potassium phosphate buffer (i.e., pH 7.0, comprising 1 mM EDTANa_2_ in addition to 0.5% PVP, *w/v*) on ice. Accordingly, centrifugation of the homogenate was conducted for 20 min at 12,000× *g* at 4 °C. The supernatant was used for the measurement of POD, SOD, APX, and CAT.

SOD activity was determined by measuring its aptitude to inhibit the photochemical reduction of nitroblue tetrazolium chloride (NBT). NBT reaction solution consisted of 50 mmol L^−1^ phosphate buffer (pH 7.8), 13 mmol L^−1^ methionine, 75 μmol L^−1^ NBT, 2 μmol L^−1^ riboflavin, and 0.1 mmol L^−1^ EDTA. The reaction began after adding 2 μmol L^−1^ riboflavin and placing the reaction tubes under 15 W fluorescent lamps for 15 min. The reaction mixture without enzyme extract was used as a control. One unit of SOD activity was elucidated as the quantity of enzyme required to cause 50% inhibition of the NBT reduction and photoreduction was observed at 560 nm [27].

The CAT activity was observed with an extermination constant of 39.4 mM^−1^ cm^−1^ at 240 nm absorbance due to the reduction of extinction H_2_O_2_. The 3 mL reaction mixture comprised of 2.8 mL phosphate buffer (25 mM, pH 7.0), 0.1 mL H_2_O_2_ (0.4%), and 0.1 mL enzyme extract. The reaction began by adding H_2_O_2_ [28] and enzyme activity was calculated in terms of l M of H_2_O_2_ g^−1^ FW min^−1^ at 25 ± 2 °C.

The POD activity was assessed using the reaction mixture consisting of 10 mM H_2_O_2_ in 50 mM Tris buffer (pH7.0) and 25 μL of enzyme extract in a total volume of 750 μL [29]. The total of 750 μL reaction solution contained 25 μL enzyme extract mixed with 1% (*v/v*) guaiacol and 0.4% (*v/v*) H_2_O_2_ assembled in 50 mM Tris buffer (pH 7.0). Changes in absorbance related to the oxidation of guaiacol (ε = 25.5 mM^−1^ cm^−1^) were calculated at 470 nm. The enzyme activity was calculated in terms of l M of guaiacol oxidized g^−1^ FW min^−1^ at 25 ± 2 °C.

The APX activity was calculated depending on the decrease in absorbance at 290 nm as ascorbate was oxidized. The 3 mL reaction mixture contained 2.7 mL phosphate buffer (25 mM, pH 7.0), 0.1 mL ascorbate (7.5 mM), 0.1 mL H_2_O_2_ (0.4%), and 0.1 mL of enzyme extract. The reaction began by adding H_2_O_2_ and the enzyme activity was calculated as μmol min^−1^ mg^−1^ protein at 25 ± 2 °C [30].

### 2.11. RNA Extraction and Gene Expression Analysis

Gene expression of antioxidant enzymes was measured through quantitative real-time PCR (qRT-PCR). Frozen shoot and root samples were ground comprehensively in liquid nitrogen using a mortar and pestle. Total RNA was extracted from both shoots and roots by Trizol mixture by following an already described method [31]. The RNA concentration and its purity were determined through NanoDrop 2000/2000 c (Thermo-Fisher Scientific, Waltham, MA, USA). For the synthesis of cDNA, the PrimeScript™ RT reagent kit was utilized. The primers used to quantify the expression of *SOD-Cu-Zn*, *SOD-Fe_2_*, *APX02, APX08, CATa, CATb, POX1,* and *POX2* genes are listed in Supplementary Table S1. The 20-μL reaction mixture was made with 2XSYBR Green Master Mix reagent (10 μL volume) (Applied Biosystems, Foster City, CA, USA), cDNA samples (6 μL volume), and 200 nM gene-specific primers. The thermocycler was set as follows: 95 °C for 3 min; 40 cycles of 95 °C for 30 s, 60 °C for 30 s, and 72 °C for 1 min. The relative change in the expression of genes was determined according to Livak et al. [32]. The *OsActin* was used for internal calibration as a control gene to normalize the other genes.

### 2.12. Statistical Analysis

Different treatments for one-way analysis of variance through the least significant differences (LSD) were pragmatic as a posthoc test at 95% assurance interlude amongst frequent data set by using SPSS v16.0 (SPSS, Inc., Chicago, IL, USA). The analysis of variance [33] was conducted via Duncan’s multiple range test amongst various treatment means to accomplish the significant difference at *p* < 0.05 and 0.01 level among mean values. The principal component analysis (PCA) and agglomerative hierarchical clustering (AHC) were accomplished to examine the classification and grouping of two different cultivars of rice for their vulnerability to Cr by using Minitab software version 18.1.

## 3. Results

### 3.1. Determination of the Significant Effect of Cr (VI) on Seed Vigor and Plant Development

**Figure 1:**
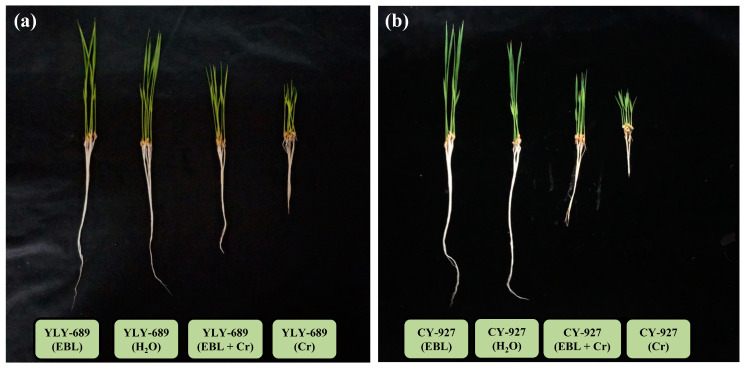
Physiological effect of Cr toxicity on two different rice cultivars. (**a**) Physiological effect of Cr on the YLY-689 cultivar and stress alleviation effect of 0.01 µM EBL under 100 µM Cr concentration. (**b**) Physiological effect of Cr on cultivar CY-927 and stress alleviation effect of 0.01 µM EBL under 100 µM Cr concentration.

Based on the preliminary experiment results, Cr toxicity caused clear phenotypic changes in both the cultivars at various concentrations (0, 50, 100, 200, 300, and 400 µM). Cr imposed higher toxicity at 200 µM and above concentrations, and hence, a 100 µM Cr concentration was selected for further experimentation based on phenotypical alterations. Brassinosteroids (0.01 µM) showed a positive effect on stress mitigation under Cr exposure on all concentrations (Figure 1).

Germination energy and the germination percentage were significantly reduced by the exposure of Cr with a concentration of 100 µM compared to control in both cultivars (Table 1). Seed germination was observed to be better in plants treated with 0.01 µM brassinosteroids (EBL) compared to seeds primed with water (H_2_O). A clear reduction in germination energy and germination percentage was observed in cultivar CY-927 under Cr stress. Germination index and vigor index were decreased in seeds primed with water in both cultivars but the mean germination time was decreased in seeds primed with EBL under Cr stress (Table 1).

Shoot length, root length, fresh weight, and dry weight were also affected by Cr toxicity in both cultivars. Shoot length and root length were significantly decreased in both cultivars under stress conditions compared to control, but more lessening was noted in cultivar CY-927. Fresh weight and dry weight were also lower under Cr stress in plants primed with water. Priming with EBL showed mitigation behavior toward Cr toxicity in both cultivars (Table 2).

### 3.2. Seed Priming with EBL Significantly Enhanced Photosynthetic Pigments under Cr Stress

**Figure 2:**
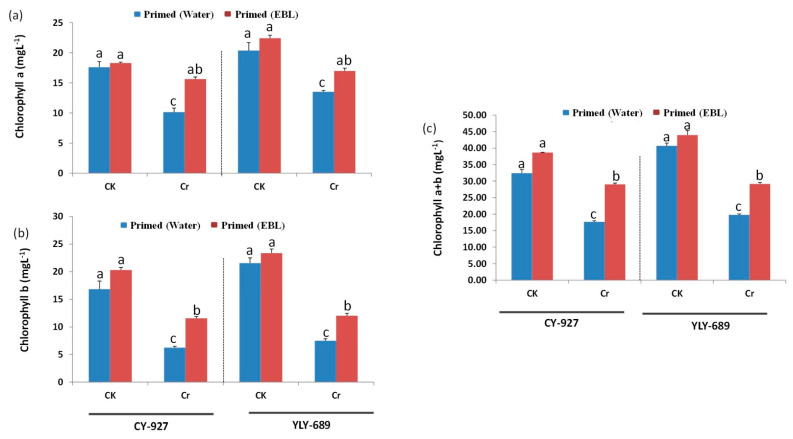
Effect of brassinosteroids on chlorophyll contents in two different rice cultivars under Cr toxicity. (**a**) Chlorophyll a contents in both rice cultivars; (**b**) Chlorophyll b contents in both rice cultivars; (**c**) Total Chlorophyll contents (Chlorophyll a+b) in both rice cultivars. The values presented are means ± SDs (*n* = 3). Different letters (a–c) above bars show a significant difference at *p* < 0.05 among treatments.

Results represented that Cr toxicity caused a clear reduction in chlorophyll a (Chla), chlorophyll b (Chlb), and total chlorophyll contents compared to the control (Figure 2). In cultivar CY-927, decreased Chla, Chlb, and total chlorophyll content was noted to be more pronounced than YLY-689 under Cr toxicity. Seed priming with EBL exhibited a significant increase in Chla, Chlb, and total chlorophyll at 100 µM Cr stress compared to seeds primed with water (Figure 2).

### 3.3. Accumulation of Cr Contents Was Reduced Significantly by EBL Seed Priming

Results demonstrated that Cr accumulation was more pronounced inside roots compared to shoots and uptake was higher in cultivar YLY-689 than CY-927 (Supplementary Tables S2 and S3). Cr uptake caused macronutrient imbalance as well. Under Cr toxicity, Mn, Cu, and Zn uptake were decreased in both cultivars; however, this decrease was more obvious in cultivar CY-927 than YLY-689 in both roots (Supplementary Table S3) as well as shoots (Supplementary Table S2).

Results demonstrated that the effect of brassinosteroids diminished the uptake and accumulation of Cr and maintained the nutrient balance in both cultivars of rice. Mn, Zn, and Cu contents were increased by EBL treatment under Cr stress in shoots and the same trend of nutrient balance was observed in roots of both cultivars (Supplementary Tables S1 and S2).

### 3.4. Significant Reduction of MDA Contents and H_2_O_2_ Production by Seed Priming with EBL

**Figure 3:**
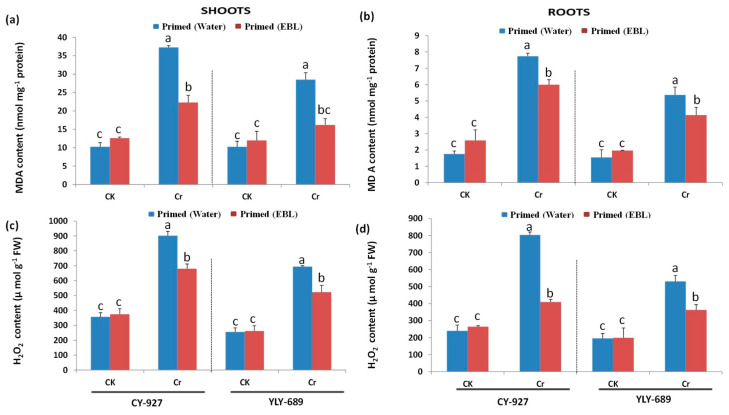
Effect of Cr toxicity on MDA contents as well as H_2_O_2_ production and alleviation behavior of EBL toward MDA contents and H_2_O_2_ production under Cr stress in both varieties of rice. (**a**) MDA contents in shoots of both rice cultivars; (**b**) MDA contents in roots of both rice cultivars; (**c**) H_2_O_2_ contents in shoots of both rice cultivars; (**d**) H_2_O_2_ contents in roots of both rice cultivars. The values presented are means ± SDs (*n* = 3). Different letters (a–c) above bars show a significant difference at *p* < 0.05 among treatments.

Lipid peroxidation was estimated in both cultivars of rice plants. Cr stress enhanced MDA contents in both roots and shoots compared to the control. MDA content was higher in YLY-689, estimated as 72.50% and 38.41% in shoots and roots, respectively, compared to CY-927 (64% and 34.60% in shoots and roots, respectively) (Figure 3). MDA contents remained pronounced in shoots of both plants. The application of EBL represented the significant decline of MDA contents in shoots and roots of both varieties (e.g., 36.90 and 15.40% for YLY-689 and 38.40 and 20% for CY-927, respectively) compared to the plants primed with water.

Recent outcomes revealed that H_2_O_2_ production was enhanced under 100 µM Cr toxicity in both cultivars as compared to the control (Figure 3). More production was recorded in cultivar YLY-689 with 58.1% and 65.2% in both shoots and roots, respectively, CY-927 with 57.3 and 62.2% individually in both shoots and roots. Seeds primed with brassinosteroids showed lower MDA contents in shoots and roots of both varieties YLY-689 and CY-927 at 45.6, 35.3, 44.8, and 46.6%, respectively, compared to control.

### 3.5. Regulation of Antioxidant Activities via Seed Priming with EBL

**Figure 4:**
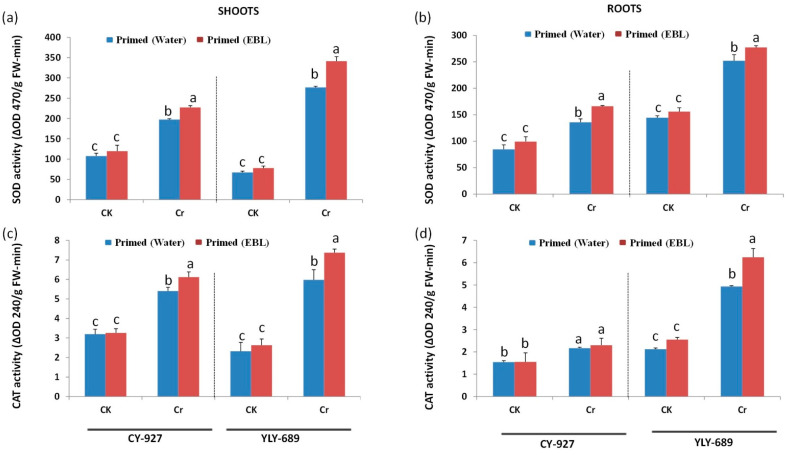

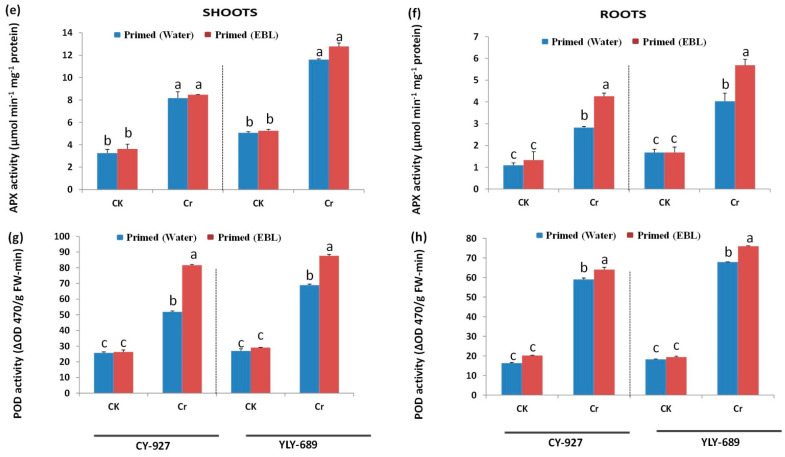
Upregulation of antioxidant enzyme activities by seed priming with EBL under Cr stress in both cultivars. (**a**) and (**b**) superoxide dismutase (SOD) in shoots and roots of both rice cultivars, respectively; (**c**,**d**) catalase in shoots and roots of both rice cultivars, respectively; (**e**,**f**) ascorbate peroxidase (APX) in shoots and roots of both rice cultivars, respectively; (**g**,**h**) peroxidase (POD) in shoots and roots of both rice cultivars, respectively. The values presented are means ± SDs (*n* = 3). Different letters (a–c) above bars show a significant difference at *p* < 0.05 among treatments.

Under Cr stress alone, antioxidant activities were modulated differently. The results validated that 100 µM Cr stress significantly enhanced the antioxidants (SOD, CAT, POD, and APX) compared to the control in both cultivars. A larger increment was observed in shoots and the same trend was noticed in both cultivars. The change was more pronounced in YLY-689 as compared to the CY-927 cultivar (Figure 4). Seed priming with 0.01 µM EBL showed very interesting behavior towards antioxidant enzyme activities and enhanced more SOD, POD, CAT, and APX under 100 µM Cr stress compared to plants primed with water. The results revealed that SOD activity was more prominent than other antioxidants in shoots of YLY-689 cultivar compared to the non-stressed plants. SOD, POD, CAT, and APX were enhanced by Cr treatment 75.7%, 64.80%, 57%, and 60.7%, respectively in shoots and 38.5%, 45.8%, 43.8%, and 48.4% in roots, respectively in YLY-689. Further, 45.3%, 56.4%, 41.3%, and 58.4% in shoots correspondingly alongside 37.8%, 20.6%, 33.1%, and 38.5% individually was enhanced in roots of CY-927 cultivars and it further enhanced by EBL together in roots and shoots of both cultivars.

### 3.6. Determination of Ultrastructure Analysis

**Figure 5:**
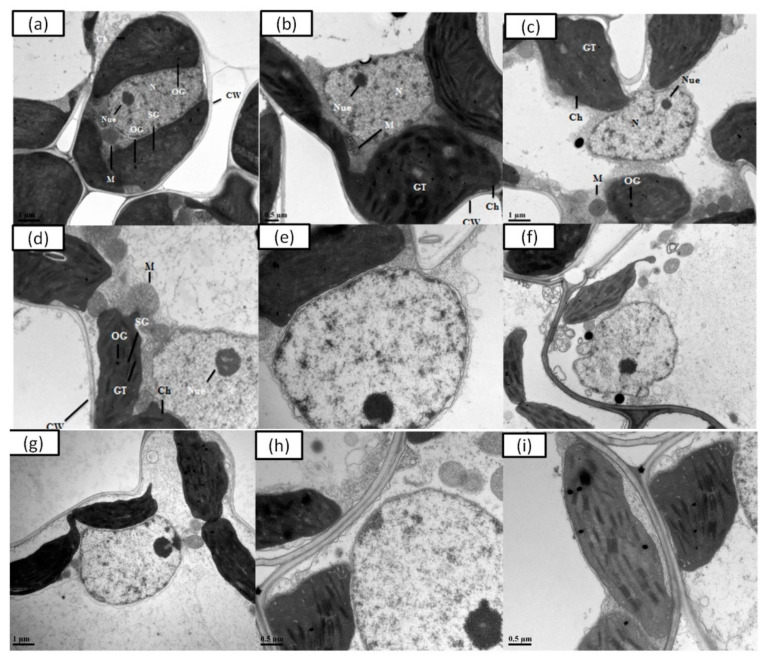
Electron micrographs of leaf mesophyll cells of two various rice cultivars (CY-927, YLY-689) primed with water as well as primed with 0.01 µM EBL grow under control and exposure of 100 µM Cr stress. (**a**) Leaf mesophyll cell of CY-927 (primed with water) at the control level. (**b**) Leaf mesophyll cell of CY-927 (primed with 0.01 µM EBL) at the control level. (**c**) Leaf mesophyll cell of YLY-689 (primed with water) at the control level. (**d**) Leaf mesophyll cell of YLY-689 (primed with 0.01 µM EBL) at the control level. (**e**) Leaf mesophyll cell of CY-927 (primed with water) underexposure of 100 µM Cr toxicity. (**f**) Leaf mesophyll cell of CY-927 (primed with 0.01 µM EBL) under 100 µM Cr toxicity. (**g**) Leaf mesophyll cell of YLY-689 (primed with water) under the disclosure of 100 µM Cr stress. (**h**,**i**) Leaf mesophyll cell of YLY-689 (primed with 0.01 µM EBL) under 100 µM Cr toxicity, N (nucleus); CW (cell wall); Ch (chloroplast); GT (granule thylakoids); M (mitochondria); Nue (nucleolus); NM (nuclear membrane).

The current study demonstrated the ultrastructural changing inside mesophyll cells of rice cultivars primed with water and with 0.01 µM EBL growing under control conditions as well as under the exposure of 100 µM Cr stress. The TEM micrographs of leaf mesophyll cells of CY-927 and YLY-689 primed with water and with 0.01 µM EBL represented normal structure with the hygienic and thin cell wall, normal organelles, as well as healthy chloroplast and granule thylakoids (Figure 5). Although, mesophyll cells of CY-927 primed with water under 100 µM Cr stress showed a damaged structure of the nucleolus as well as a double-layered nuclear membrane expansion (Figure 5). Comparatively, mesophyll cells of CY-927 primed with 0.01 µM EBL showed slight damage in nucleolus development; however, the chloroplast was normal compared to the mesophyll cells of CY-927 primed with water (Figure 5). A nuclear membrane was also observed normally in mesophyll cells of CY-927 primed with 0.01 µM EBL.

The TEM micrographs of leaf mesophyll cells of YLY-689 primed with water as well as primed with 0.01 µM EBL in control plants showed normal growth with a healthy structure of organelles (Figure 5). Whereas, mesophyll cells of YLY-689 primed with water under 100 µM Cr stress showed damage in chloroplast development and granule thylakoids, which were thinner compared to the control. In addition, the nucleolus was also observed to be abnormal (developed double nucleolus) (Figure 5). On the other hand, the mesophyll cells of YLY-689 primed with 0.01 µM EBL demonstrated better chloroplast development with little damage to thylakoid development. Furthermore, it comprised normal cell arrangements as well as the normal development of the nucleolus besides the nuclear membrane (Figure 5).

### 3.7. Determination of Gene Expression Analysis

The expression of *SOD-Cu-Zn* and *SOD-Fe2* was significantly higher in seeds primed with water under Cr toxicity compared with control. Moreover, expression was increased in seeds primed with 0.01 µM EBL compared to seeds primed with water in both cultivars (Supplementary Figure S1). The transcription level was higher in the YLY-689 cultivar compared to CY-927 (*p* < 0.01). Gene expression was noted to be higher in shoots of both cultivars than roots.

Moreover, the transcription level of genes *APX02* and *APX08* were observed to be high in both shoots and roots of the CY-927 cultivar under Cr stress. Nevertheless, significant upregulation was observed in shoots of CY-927 compared to roots (Supplementary Figure S2). Furthermore, an upregulation in the expression of both genes was noted in seedlings of plants primed with 0.01 µM EBL compared to seeds primed with water under Cr toxicity. Interestingly, the behavior of cultivar YLY-689 was quite different from CY-927 in terms of gene expression. A significant downregulation of genes *APX02* and *APX08* was calculated in roots and shoots under Cr stress. However, expression was higher in plants primed with 0.01 µM EBL compared to plants primed with water but this increment was non-significant (Supplementary Figure S2).

The same trend in transcription level of both *CATa, CATb* genes was observed. A significant increase was observed in gene expression of *CATa* and *CATb* in CY-927 for both shoots and roots, but shoots represented a higher transcription level compared to roots (Supplementary Figure S3). Expression was more prominent in seeds primed with 0.01 µM EBL compared to seeds primed with water. Downregulation was observed in both shoots and roots of cultivar YLY-689 under Cr stress. Seeds primed with 0.01 µM EBL showed a significantly enhanced transcription level compared to seeds primed with water under Cr stress conditions (Supplementary Figure S3).

A significant upregulation of genes *POX1* and *POX2* were measured in both roots and shoots under 100 µM Cr concentration in both rice cultivars. Significant upregulation of *POX2* was noted in shoots of the CY-927 cultivar under Cr stress. In both cultivars, data represented that the transcription level of *POX1* and *POX2* was significantly enhanced in plants primed with 0.01 µM EBL, and this trend was found to be similar to the results of antioxidant enzymatic activity under Cr stress. Furthermore, upregulation was more obvious in shoots of both cultivars than roots (Supplementary Figure S4).

### 3.8. Determination of Interaction among Growth and Physiological Parameters through Principal Component Analysis, Clustering, and Correlation Analysis

**Figure 6:**
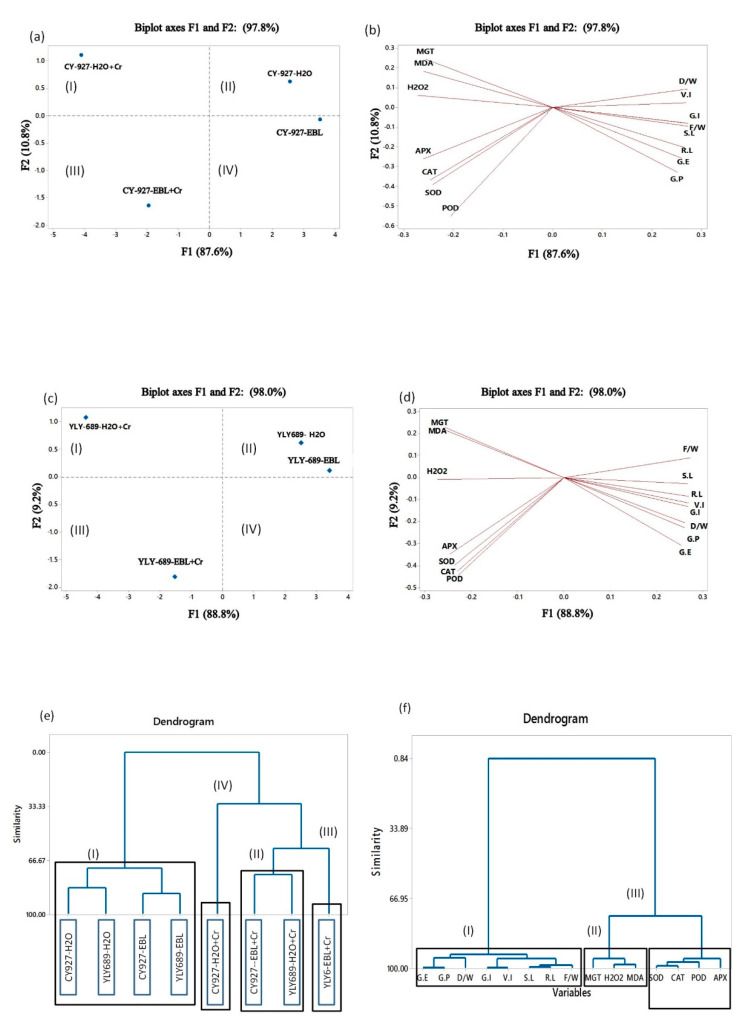
Bioplot of principle components 1 and 2 of the PCA extracted from the results obtained from physiological data of two various rice cultivars (CY927, YLY689) under different treatments such as control primed with water (CY927-H_2_O, YLY689-H_2_O), control primed with EBL (CY927-EBL, YLY689-EBL), seed primed with EBL, and treatment under Cr stress (CY927-EBL + Cr, YLY689-EBL + Cr), seed primed with H_2_O under Cr stress (CY92-Cr + H_2_O, YLY689-Cr + H_2_O). A sharp angle represented a positive correlation, an obtuse angle showed a negative correlation, and a right angle demonstrated a correlation between parameters. (**a**) Representation of the correlation between different treatments in rice cultivar CY927. (**b**) Physiological parameters of rice variety CY927 are represented through Pearson’s correlation coefficients under different treatments. (**c**) Illustration of correlation between various treatments in rice cultivar YLY689. (**d**) Physiological parameters of rice variety YLY689 representation via Pearson’s correlation coefficients under various treatments. Distance between each line represented the strength of the correlation. (I) contains MDA, MGT, and H_2_O_2_; (II) shows G.I, F/W, D/W, G.E, G.P, and V.I; (III) illustrates POD, CAT, APX, and SOD; (IV) represents R.L and S.L. (**e**) Dendrogram of two different rice cultivars under various treatments obtained through agglomerative hierarchical clustering using Ward’s method based on physiological traits. (**f**) The dendrogram demonstrated a correlation between various physiological parameters of two different rice cultivars under numerous treatments obtained through agglomerative hierarchical clustering using Ward’s method.

Principle component analysis presented the interaction of measured growth and physiological parameters under the influence of different treatments in both varieties (Figure 6). In the PCA analysis, it was found that there was a close interrelation among MDA, MGT, and H_2_O_2_. Although, MGT, H_2_O_2_, and MDA showed a significantly negative correlation with V.I, F/W, D/W, S.L, R.L, G.E, and G.P demonstrated a negative correlation with SOD, POD, CAT, and APX as well (Figure 6). It demonstrated the maximum contribution of F1 (87.6%) followed by F2 (10.80%) with a total contribution of 97.80% in CY927 and for YLY689 the maximum contribution of F1 (88.8%) followed by F2 (9.2%), with a total contribution of 98.0% was noticed (Figure 6). ACH outcomes also confirmed both varieties’ responses under distinct treatments (Figure 6). The dendrogram represented three groups that characterized the close correlation between both cultivars (CY927 and YLY689) primed with EBL as well as primed with water under Cr stress. Cultivars primed with EBL under Cr stress showed a close correlation with both controls (primed with water and EBL) compared to plants primed with water under Cr toxicity (Figure 6). The dendrogram showed the same pattern of treatments as demonstrated by PCA (Figure 6).

## 4. Discussion

### 4.1. EBL Improve Physio-Biochemical Effects Caused by Cr Toxicity in Rice Plants

Recently, increasing industrialization has become a serious threat to soil contamination which is now the main source of plant growth inhibition by disturbing various mechanisms of plants at the physiological and molecular level [34]. This study examined that chromium caused plant growth inhibition and induced negative physio-biochemical processes in two rice varieties. Reportedly, plants develop various mechanisms to overcome stress by inducing antioxidant activities, biochemical mechanisms, phytochelatins, and various hormones [35].

In the current research, the role of brassinosteroids (EBL) has been elaborated to overcome Cr toxicity. Cr has reduced the plant biomass, altered the structure of roots, and affected seed germination energy, percentage, vigor index, and mean germination time. Seed germination was affected because of heavy metal toxicity through the access of rice plants’ embryonic tissues and due to the structure of seed coats. When a seed radicle comes into contact with Cr; it lowers the germination rate by lowering the α and β amylase activities, reducing the sugar supply to the seeds, and constrain the seed germination rate [36]. Root and shoot length also reduce under Cr stress and it may be caused by nutrient uptake disturbance inside rice plants and may decrease the cell division and elongation.

The root is the primary point of contact with Cr; however, the toxicity and accumulation of Cr hinder root elongation in plants. Cr uptake might be caused by the imbalance of nutrients such as Zn, Mn, and Cu, which is necessary to regulate various plant growth mechanisms. The current study revealed that the application of EBL caused a positive effect on plant growth and development under Cr stress by reducing the uptake of Cr and by enhancing nutrient contents (Zn, Cu, and Mn) significantly in both rice cultivars. This has been translated to improve shoot length, root length, and fresh and dry weight of plants under Cr stress. It also maintained the germination rate in rice cultivars by enhancing cell division and elongation processes [37]. Similar studies have been focused on various plant species such as *Raphanus sativus* [38], *Hordeum vulgaris* [39], and *Lycopersicon esculentum* [40].

### 4.2. EBL Prevented Degradation of Chlorophyll Pigments under Cr Stress

This research showed that Cr caused a significant reduction in Chla, Chlb, and total chlorophyll contents (Figure 2) in both rice varieties. Degradation of chlorophyll is the primary sign of Cr toxicity, which is the leading indicator of phytotoxicity in plants [41]. The reduction of photosynthetic pigments might result from ROS activity enhancement under Cr toxicity [42]. In this study, seed priming with EBL showed a significant increment in photosynthetic pigments under Cr toxicity as compared to plants primed with water. Based on previous studies, CO_2_ acclimatization was augmented by EBL, and it enhanced some specific genes’ expression to increase the antioxidant enzyme activities and to scavenge ROS activity [36].

### 4.3. Seed Priming with EBL Reduced MDA Contents as Well as H_2_O_2_ Production

The results revealed that there is a higher production of H_2_O_2_ and MDA contents under Cr stress compared to the control plants (Figure 3). ROS activity was relatively higher in shoots compared to roots. Previous studies also indicated that *Arabidopsis Thaliana* exposed to heavy metals and rice caused an increase in ROS activity [43,44]. The higher ROS concentration causes oxidative damage in biomolecules, such as DNA, RNA, proteins, and pigments in addition to lipid peroxidation [45]. In this study, seeds primed with EBL reduced ROS activity under Cr stress by protecting membrane damage as compared to seeds primed with water. Moreover, EBL also protected mung bean plants from membrane damage by reducing ROS activity. EBL also proved this protective behavior towards the green bell pepper under chilling stress by reducing MDA contents [46]. Besides, EBL foliar spray in tomato plants decreased oxidative membrane damage and lipid peroxidation [47]. EBL enhanced the resistance capability of various plants by reducing ROS activity because of enhancing antioxidant activity to scavenge ROS accumulation in plants such as the mung bean, soybean, pea epicotyls, bean, sunflower, and cucumber hypocotyls, *Arabidopsis* peduncles, and *Hordeum vulgare* [48,49,50]. Similarly, a recent study demonstrated the protective behavior of EBL towards Zn-induced oxidative damage in *Solanum nigrum* L. plants [51]. In another study, Jan et al. [12] reported that EBL significantly reduced the Cr toxicity in tomato plants and also improved the growth, physio-biochemical attributes, and antioxidant activity. It also has been proven that EBL mitigated the lead toxicity in *Brassica juncea* L. by scavenging ROS activity [52].

### 4.4. EBL Enhanced Antioxidant Activity to Mitigate Cr Toxicity

An increase in MDA contents and H_2_O_2_ production causes oxidative and lipid peroxidation damage inside plants and disturbs the metabolic processes, function, and structure of membranes [53]. As a result, physiological processes besides growth inhibition also occur in rice seedlings. To scavenge the higher production of MDA and H_2_O_2_, the antioxidant defense mechanism (SOD, POD, CAT, and APX) is stimulated in plants [54,55]. In a recent study, Khan et al. [56] revealed that EBL significantly increased plant growth and triggered the antioxidant defense system in wheat plants against drought stress. Similarly, Rattan et al. [57] reported that EBL alleviated salt stress in maize seedlings by regulating the antioxidant enzyme activities. Another study revealed that EBL detoxified the combined toxicity of salinity and potassium deficiency in barley plants by modulating the antioxidant defense mechanism [58], and enhanced manganese tolerance in *Arabidopsis thaliana* L. by regulating the antioxidant defense mechanism [59].

### 4.5. Rice Cultivar YLY-689 Was More Resistant to Cr Stress Than CY-927

The biomass (fresh and dry weight, and shoot and root length), germination energy, germination percentage, germination index, and vigor index were reduced while mean germination time was increased in CY-927 YLY-689 cultivar under Cr toxicity (Table 1 and Table 2). These results further indicated the EBL role in mitigating Cr toxicity in rice plants in both sensitive and tolerant varieties. Cr caused a decrease in the growth of plant roots and shoots in addition to the height [55,60,61]. Likewise, Cr instigated a reduction in biomass and some other seed germination parameters (Table 1 and Table 2). Nonetheless, YLY-689 showed more resistance toward Cr stress compared to CY-927.

### 4.6. Ultrastructural Changing Induced by Cr in Rice Plants

The influence of 100 µM Cr toxicity on the ultrastructure of leaf mesophyll cells was observed in both cultivars. Outcomes demonstrated that both rice cultivars (CY-927 and YLY-689) primed with water and with 0.01 µM EBL had well-developed chloroplast, granule thylakoids, nucleolus, nuclear membrane, cell wall, mitochondria as well as thylakoid membranes (Figure 5). The plants under exposure of Cr (100 µM) showed ruptured chloroplasts with damaged granule thylakoids (Figure 5) besides having an abnormal nucleolus with nuclear membrane aberrations (Figure 5). Similar outcomes were observed in rice and *Lolium perenne* L. [62,63]. The reduction in chlorophyll contents occurred because of the inhibition of specific enzyme biosynthesis involved in chlorophyll content productions. Cr accrued inside cells and caused damage inside chloroplasts by producing abnormalities in granule thylakoids through swollen chloroplasts. It directly affected the photosynthesis rate under a stressed condition. The thylakoid membrane ruptured because of Cr toxicity in *Brassica napus* L. and inevitably changed chloroplast structure [64].

There is a close relationship between the structure and function of rice seedlings. However, the Cr impact on chloroplasts has an essential role in physiological alternations in plants. Chloroplast aberrations also have a role in the reduction of chlorophyll contents. The decrease in chlorophyll contents and photosynthetic pigments mainly caused a reduction in photosynthetic rate [65].

In the current study, both cultivars primed with 0.01 µM EBL showed resistance towards Cr toxicity. In comparison with the control, less damage was observed in chloroplasts and granule thylakoids compared to the control under Cr toxicity. Nucleolus and nuclear membrane aberrations were also lower in plants primed with EBL than plants primed with water and exposed to Cr toxicity. Hence, it was found that priming with EBL has positively affected both cultivars and played its role in maintaining chlorophyll contents in rice plants.

### 4.7. Gene Expression Level

The expression level of genes was investigated at the mRNA level in both shoots and roots of the rice seedlings of both cultivars. The estimation of antioxidant gene expression together with antioxidant enzyme activities presented a better evaluation of these measurements after priming with EBL. It was investigated that Cr toxicity is mitigated in rice plants because of the upregulation of SOD, APX, CAT, and POD activities [65,66]. In our study, SOD and POD gene expression was observed to be upregulated in both cultivars. Whereas upregulation of the transcription level of CAT and APX genes was observed in cultivar CY-927. The same trend of gene expression level was observed in various plants such as *Raphanus Sativa* [38], *Cicer arietinum* [67], and Tomato [12]. Additionally, SOD, POD, CAT, and APX activities increased in plants treated with EBL under Cr toxicity in both cultivars, and the upregulation of their genes was noted with the same pattern. The increase in antioxidant activities and transcription level of antioxidant activity-related genes was due to the accretion of salicylic acid contents through BR signaling pathways. Furthermore, it mitigates the Cr toxicity and enhances the tolerance mechanism inside plants against heavy metal toxicity. Interestingly, in our findings, APX and CAT transcriptional levels in both roots and shoots of the YLY-689 cultivar were quite the opposite of cultivar CY-927. It also varied from the results of antioxidant enzyme activities. Although, gene expression of SOD and POD showed the same trend with antioxidant enzyme activities in both rice cultivars. The distinction between APX and CAT gene expression with its enzymatic activities was also investigated in *Brassica napus* [64] and cotton [55] plants as well under Cr stress. The discrepancy between antioxidant enzyme activities and gene expressions under Cr toxicity demonstrated that it might have some vital role inside the plant defense system against Cr stress mitigation.

### 4.8. Clustering and Correlation Analysis

We used principal component analysis (PCA) to recognize and categorize the large dataset in terms of growth and physiological parameters into a small number of dynamically interrelated variables in our study [68,69]. It was found that EBL priming of rice seedlings in both cultivars was distinctly separate in PCA compared to Cr stressed plants. This placement of EBL-primed plants in separate coordinates compared with Cr-stressed plants was more prominent in the case of variety CY-927 (Figure 6) than variety YLY-689 (Figure 6). This disclosed the interaction between different genotypes of rice based on distinct treatments (Figure 6). On the basis of physiological characteristics, different treatments were exploited to discriminate the sensitive and tolerant genotype besides representing the correlation among various traits by using the amalgamation of both PCA and ACH (Figure 4). V.I, F/W, D/W, S.L, R.L, G.E, and G.P showed to be a group with a significantly positive correlation between each other but instantaneously negative relation with MGT, H_2_O_2_, and MDA.

## 5. Conclusions

Chromium toxicity caused adverse effects on rice plants’ physiological, biochemical, and molecular mechanisms, which negatively affected seed germination parameters and further reduced the plants’ growth and development. Seed priming with brassinosteroids (EBL) improved seed germination attributes and capped the worst effect of Cr on both cultivars of rice plants by triggering its physio-biochemical processes such as through maintaining chlorophyll contents, increasing mineral uptakes via reducing Cr uptake and accumulation, by enhancing antioxidant enzyme activities, as well as lessening ROS production under Cr stress. Furthermore, EBL improved the ultrastructure of both cultivars of rice under Cr toxicity. Our studies ensured the competency of EBL to cope and detoxify the nastiest effects of Cr in rice plants. Moreover, CY-927 was more affected by Cr stress rather than the YLY-689 cultivar. Thus, the YLY-689 cultivar is more tolerant toward Cr stress.

## Figures and Tables

**Table 1:** Effect of Cr toxicity on the seed germination parameter in seeds primed with EBL compared to seeds primed with water in two different rice cultivars.

Varieties Name	Treatment	G.E	G.P	G.I	MGT	V.I
CY-927	H_2_O	90.00 ± 2.00 ^b^	94.67 ± 1.15 ^b^	20.13 ± 1.17 ^b^	2.91 ± 0.17 ^b^	2.04 ± 0.11 ^b^
	EBL	96.00 ± 2.00 ^a^	99.33 ± 1.15 ^a^	28.01 ± 1.47 ^a^	2.16 ± 0.11 ^c^	2.87 ± 0.13 ^a^
	H_2_O+Cr	38.67 ± 3.06 ^d^	46.67 ± 3.06 ^d^	7.15 ± 0.74 ^d^	4.06 ± 0.07 ^a^	0.31 ± 0.07 ^d^
	EBL+Cr	72.67 ± 3.06 ^c^	82.67 ± 3.06 ^c^	12.90 ± 0.35 ^c^	3.21 ± 0.14 ^b^	0.77 ± 0.03 ^c^
YLY-689	H_2_O	93.33 ± 2.31 ^a^	99.33 ± 1.15 ^a^	27.29 ± 0.76 ^b^	2.44 ± 0.13 ^c^	2.63 ± 0.09 ^b^
	EBL	98.00 ± 2.00 ^a^	100.00 ± 0.00 ^a^	32.98 ± 0.59 ^a^	2.07 ± 0.08 ^d^	3.24 ± 0.05 ^a^
	H_2_O+Cr	54.00 ± 2.00 ^c^	66.00 ± 2.00 ^c^	12.26 ± 0.88 ^d^	3.66 ± 0.19 ^a^	0.90 ± 0.07 ^d^
	EBL+Cr	86.67 ± 1.15 ^b^	89.33 ± 1.15 ^b^	21.50 ± 0.30 ^c^	2.74 ± 0.14 ^b^	2.00 ± 0.03 ^c^

Each treatment value represents the mean of three replicates ± standard deviation. Same letters are representing no significant differentiation at 95% probability level (*p* < 0.05). The data presented here represent the selected Cr concentration (100 µM). Here, germination energy (G.E); germination percentage (G.P); germination index (G.I); mean germination time (MGT); vigor index (V.I); brassinosteroids (EBL).

**Table 2:** Improvement in shoot length, root length, and fresh and dry weight by seed priming with EBL compared to the control under Cr toxicity.

Varieties Name	Treatment	S.L	R.L	F/W	D/W
CY-927	H_2_O	15.51 ± 0.02 ^b^	13.04 ± 0.02 ^b^	0.90 ± 0.01 ^b^	0.10 ± 0.001 ^b^
	EBL	17.19 ± 0.04 ^a^	15.03 ± 0.16 ^a^	0.96 ± 0.01 ^a^	0.10 ± 0.001 ^a^
	H_2_O+Cr	8.30 ± 0.03 ^d^	7.56 ± 0.03 ^d^	0.51 ± 0.01 ^d^	0.05 ± 0.001 ^d^
	EBL+Cr	11.59 ± 0.02 ^c^	10.96 ± 0.02 ^c^	0.68 ± 0.01 ^c^	0.06 ± 0.001 ^c^
YLY-689	H_2_O	15.16 ± 0.08 ^b^	13.31 ± 0.02 ^b^	0.91 ± 0.01 ^b^	0.10 ± 0.003 ^a^
	EBL	18.39 ± 0.06 ^a^	15.28 ± 0.04 ^a^	0.96 ± 0.01 ^a^	0.10 ± 0.001 ^a^
	H_2_O+Cr	9.34 ± 0.04 ^d^	8.43 ± 0.02 ^d^	0.59 ± 0.01 ^d^	0.07 ± 0.002 ^c^
	EBL+Cr	12.22 ± 0.04 ^c^	11.22 ± 0.03 ^c^	0.68 ± 0.01 ^c^	0.09 ± 0.001 ^b^

Each treatment value represents the mean of three replicates ± standard deviation. Same letters represent no significant difference at the 95% probability level (*p* < 0.05). Here, shoot length (S.L); root length (R.L); fresh weight (F/W); dry weight (D/W); brassinosteroids (EBL).

## Data Availability

The data presented in this study are available within the article.

## Supplementary Materials

The following are available online at https://www.mdpi.com/article/10.3390/antiox10071089/s1.

## Abbreviations

Cr	Chromium
CK	Control
Chla	Chlorophyll a
Chlb	Chlorophyll b
Chl a+b	Chlorophyll a+b
MDA	Malondialdehyde
H_2_O_2_	Hydrogen peroxide
SOD	Superoxide dismutase
CAT	Catalase
APX	Ascorbate peroxidase
POD	Peroxidase
G.E	Germination energy
G.P	Germination percentage
G.I	Germination index
V.I	Vigor index
MGT	Mean germination time
S.L	Shoot length
R.L	Root length
F/W	Fresh weight
D/W	Dry weight
