# Supplementary material for: Seed Priming with Brassinosteroids Alleviates Chromium Stress in Rice Cultivars via Improving ROS Metabolism and Antioxidant Defense Response at Biochemical and Molecular Levels
Source: Antioxidants (Basel). 2021 Jul 7;10(7):1089. doi: 10.3390/antiox10071089 (PMC8301181; doi:10.3390/antiox10071089)
Supplement: Supplementary file 1 [file antioxidants-10-01089-s001.zip › antioxidants-1207332-supplementary.pdf]

Supplementary Materials

Table S1. Primers information

Locus	Primer names	Primer sequences
LOC_4328073-F	CATa-1 (F)	GGAAGCTGTTCGTCCAGGTGAT
LOC_4328073-R	CATa-2 (R)	TCCGGCCATGTCTTGGTGTC
LOC_4342124-F	CATb-1 (F)	TTCTCCAGCGGTGGGTTGAT
LOC_4342124-R	CATb-2 (R)	CCAAGGGACGCATCACACTG
LOC_4340091-F	SOD-Fe_2 (F)	AGAAAAGATGGCGGCTTTC
LOC_4340091-R	SOD-Fe_2 (R)	CCCTGCCTTTGTTCTCTCG
LOC_4346329-F	SOD-Cu-Zn (F)	GGACTTACTCCTGGACTTCACG
LOC_4346329-R	SOD-Cu-Zn (R)	GTTTGGGTTAAAATGTGGTCCT
LOC_4333919-F	OsActin-1(F)	CTTCATAGGAATGGAAGCTGCGGGTA
LOC_4333919-R	OsActin-2 (R)	CGACCACCTTGATCTTCATGCTGCTA
LOC_Os07g0694700-F	OsAPX02 (F)	ACCTGAGGTCCCCTTCCAT
LOC_Os07g0694700-R	OsAPX02 (R)	CCTGCCTTAGGTGGTCAGAA
LOC_Os02g0553200-F	OsAPx08(F)	GTGTGCCCGCTGATCATCTTA
LOC_Os02g0553200-R	OsAPx08 (R)	TTTCCCCAGCCACTCCTGTCA

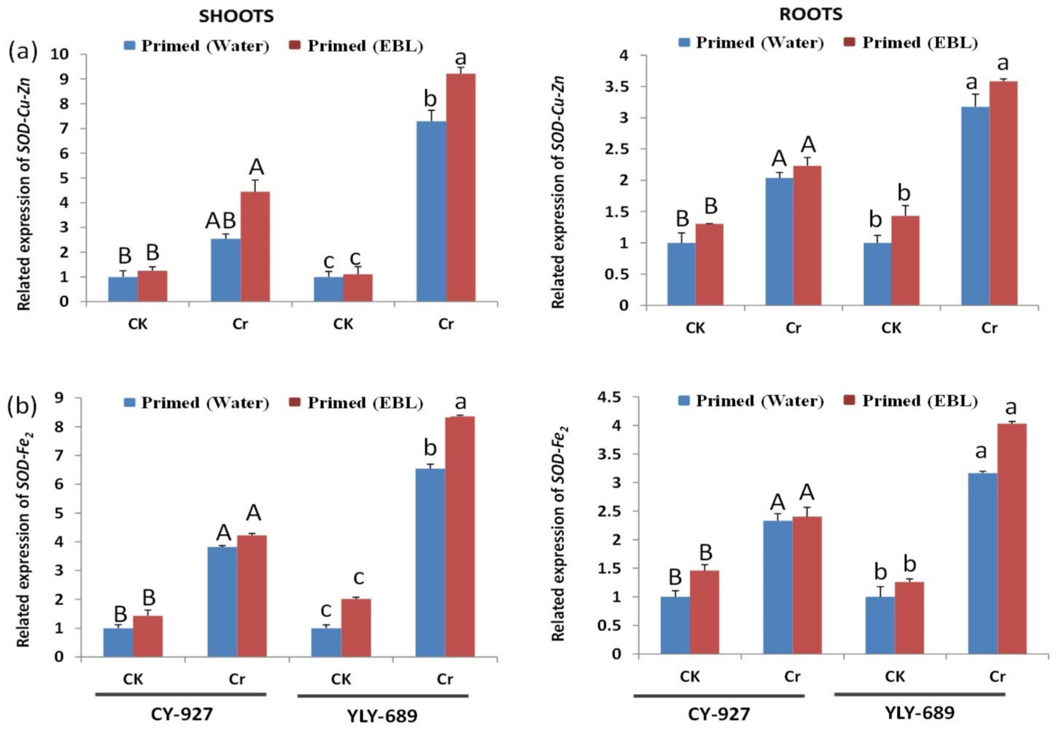

Figure S1. Gene expression of (a) *SOD-Cu-Zn* and (b) *SOD-Fe*$_2$ in both rice cultivars; primed with water as well as primed with 0.01μM EBL under 100μM Cr toxicity. (A) Control (CK); (B) Chromium (Cr). The values presented are means ± SDs (n = 3). Different letters above bars show significant difference at $p < 0.05$ among treatments.

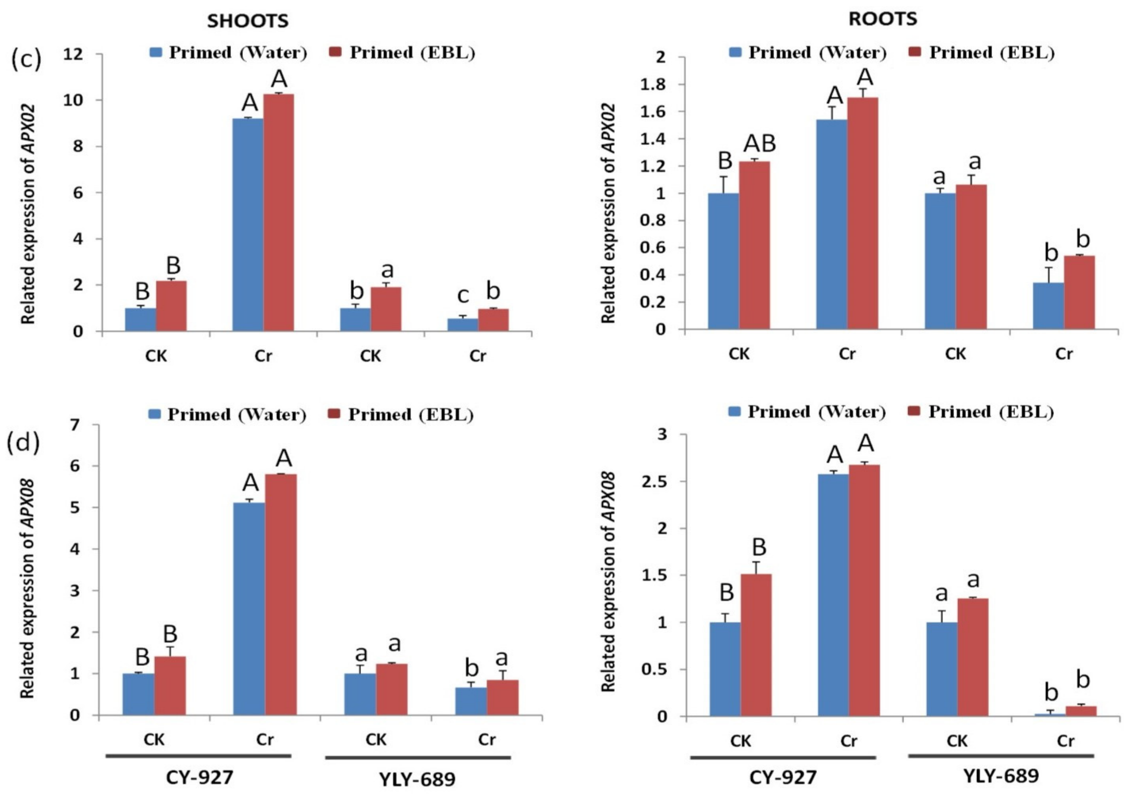

Figure S2. Effect of seed priming with 0.01μM EBL as compared to seed priming with water on gene expression of (c) *APX02* and (d) *APX08* under toxicity of Cr (100μM) concentration on both rice cultivars**.** (A) Control (CK); (B) Chromium (Cr). The values presented are means ± SDs (n = 3). Different letters above bars show significant difference at $p < 0.05$ among treatments.

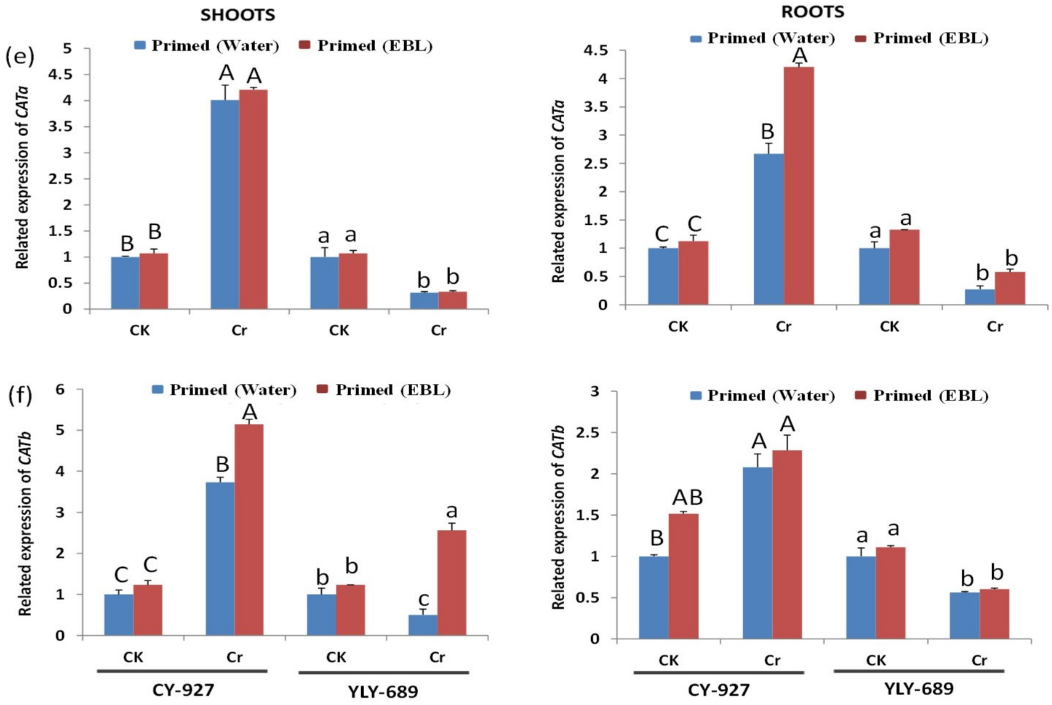

Figure S3. Effect of seed priming with 0.01μM EBL on gene expression of (e) *CATa*, (f) *CATb* in shoots and roots of both cultivars of rice under toxicity of 100μM Cr. (A) Control (CK); (B) Chromium (Cr). The values presented are means ± SDs (n = 3). Different letters above bars show significant difference at $p < 0.05$ among treatments.

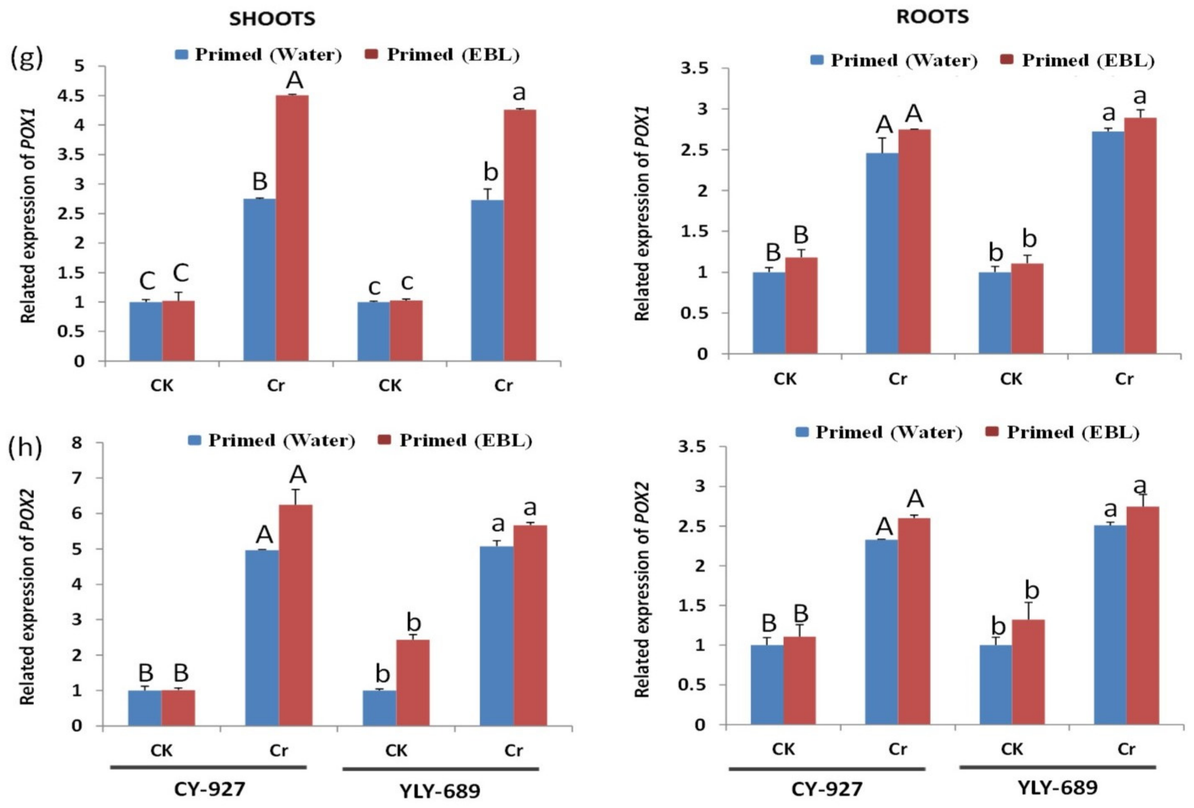

Figure S4. Effect of seed priming with 0.01μM EBL on transcription level of (g) *POX1*, (h) *POX2* under 100μM Cr stress in both rice cultivars**.** (A) Control (CK); (B) Chromium (Cr). The values presented are means ± SDs (n = 3). Different letters above bars show significant difference at $p < 0.05$ among treatments.

Table S2. Cr uptake and accumulation in shoots with or without effect of seed priming with EBL (0.01μM) in both rice cultivars.

Treatment	Cr	Mn	Cu	Zn
	mg/g	mg/g	mg/g	mg/g
CY-927-H_2O	-	1.56±0.02[a]	0.05±0.02[a]	0.24±0.09[a]
CY927-EBL	-	1.14±0.02[a]	0.04±0.01[ab]	0.19±0.02[ab]
CY-927- H_2O+Cr	0.38±0.06[a]	0.14±0.05[b]	0.02±0.01[c]	0.08±0.02[c]
CY927-EBL+Cr	0.24±0.06[b]	0.26±0.07[b]	0.03±0.01[ab]	0.14±0.03[bc]
YLY-689-H_2O	-	1.36±0.02[a]	0.04±0.01[a]	0.16±0.05[a]
YLY-689-EBL	-	1.06±0.09[a]	0.09±0.01[a]	0.15±0.04[a]
YLY-689- H_2O+Cr	0.52±0.03[a]	0.24±0.06[b]	0.03±0.01[a]	0.11±0.02[a]
YLY-689-EBL+Cr	0.35±0.05[b]	0.27±0.02[b]	0.03±0.01[a]	0.12±0.03[a]

(A) Chromium (Cr); (B) Manganese (Mn); (C) Copper (Cu) (D) Zinc (Zn). Each value is demonstrating the mean of three replicates of every treatment. Same letters are representing no significant differentiation at 95% probability level ($p<0.05$.).

Table S3. EBL application effect on Cr uptake and accumulation as well as macronutrient balance in roots of both cultivars of rice.

Treatment	Cr	Mn	Cu	Zn
	mg/g	mg/g	mg/g	mg/g
CY-927-H_2O	-	0.25±0.06[a]	0.07±0.04[a]	0.17±0.05[ab]
CY927-EBL	-	0.15±0.06[a]	0.08±0.05[a]	0.19±0.02[ab]
CY-927- H_2O+Cr	2.95±0.04[a]	0.09±0.02[ab]	0.04±0.01[ab]	0.19±0.07[ab]
CY927-EBL+Cr	2.09±0.04[b]	0.18±0.01[a]	0.08±0.02[a]	0.37±0.40[a]
YLY-689-H_2O	-	0.32±0.02[a]	0.12±0.08[a]	0.20±0.11[a]
YLY-689-EBL	-	0.31±0.09[a]	0.12±0.03[a]	0.16±0.05[ab]
YLY-689- H_2O+Cr	3.55±0.48[a]	0.05±0.03[b]	0.03±0.01[b]	0.06±0.04[b]
YLY-689-EBL+Cr	1.86±0.74[b]	0.12±0.04[ab]	0.09±0.02[ab]	0.14±0.02[ab]

(A) Chromium (Cr); (B) Manganese (Mn); (C) Copper (Cu) (D) Zinc (Zn). Each value is demonstrating the mean of three replicates of every treatment. Same letters are representing no significant differentiation at 95% probability level ($p<0.05$).
